# Perivascular Epithelioid Cell Tumour with Intraorbital Location: Report of a Case and Review of the Literature

**DOI:** 10.1155/2016/1936421

**Published:** 2016-01-26

**Authors:** Idania Lubo, Ileana Fermín, Olindo Massarelli, Roberta Gobbi, Paolo Cossu Rocca

**Affiliations:** ^1^Anatomy and Pathology Institute “Dr. José Antonio O'Daly”, Central University of Venezuela, Los Chaguaramos 1040, Caracas, Venezuela; ^2^Maxillofacial Surgery Unit, University of Sassari, Italy; ^3^Department of Histopathology and Anatomical Pathology, University of Sassari, Italy

## Abstract

The Perivascular Epithelioid Cell tumours (PEComas) are rare mesenchymal neoplasms recognized as entity by the World Health Organization. The tumour cells have an uncertain origin and are characterized by distinctive histological and immunohistochemical features. We report a case of PEComa occurring as intraorbital lesion in a 47-year-old man. We found only two other cases described in the literature and we considered all three cases together in order of histology, immunohistochemistry, and clinical outcome. We found a strict histological overlapping and quite similar immunohistological results. All three cases showed a favourable clinical course probably related to small size of tumours (<5 cm), low mitotic rate (<2 mitoses in 50 HPF), and absence of necrosis.

## 1. Introduction

Bonetti et al. [1] firstly proposed the descriptive name of Perivascular Epithelioid Cells (PEC) in 1992, whereas Zamboni et al. [2] subsequently introduced the term PEComa in 1996. The Perivascular Epithelioid Cell tumours (PEComas) represent a family of rare mesenchymal neoplasms, including angiomyolipoma (AML), clear cell “sugar” tumour (CCST) of the lung, lymphangioleiomyomatosis (LAM), clear cell myomelanocytic tumour (of the falciform ligament/ligament teres), and unusual clear cell tumours of the pancreas, rectum, abdominal serosa, uterus, vulva, thigh, and heart [3]. The tumour is more frequent in females than in males (6 : 1) with a median age of 45 years. Some cases are associated with the Tuberous Sclerosis Complex, especially AML, CCST, and LAM [3].

Although the tumour cells have an uncertain origin, they however display histological and immunohistochemical distinctive features. Owing to the variety of condition of origin, the histological pattern is not uniform, the constant features being represented by a nested architecture composed of epithelioid cells with abundant eosinophilic or clear granular cytoplasm typically surrounded by thin capillary vessels. On immunohistochemical ground the cells characteristically express HMB45 but are negative for S100 protein.

The site of origin of this tumour is extremely variable and has been reported in kidney, bladder, prostate, uterus, ovary, vulva and vagina, lung, pancreas, liver, GIT, nasal cavity, soft tissues, retroperitoneum, and bone [4].

Orbital location is exceptionally rare. At the best of our knowledge, up to date, only 2 other cases have been described in the literature [5, 6]. We present a case of intraorbital Perivascular Epithelioid Cell neoplasm along with a comparison of the 2 previously reported cases.

## 2. Case Presentation

A 47-year-old male patient complained of progressive diplopia lasting 4 months. Otherwise the patient was in a healthy condition. Ophthalmological examination revealed a marked hypofunction of the left inferior oblique muscle.

**Figure 1:**
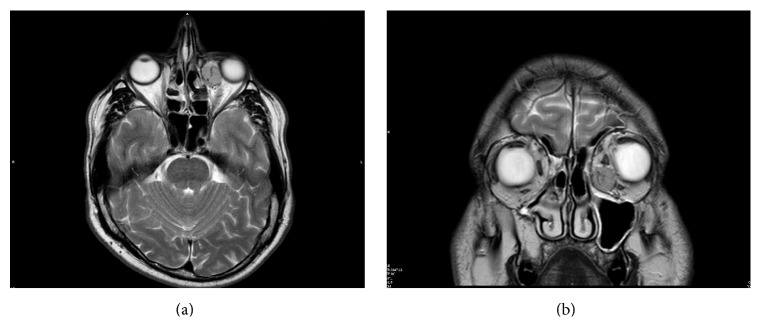
RMI: (a) Coronal T2 MRI scan shows an oval, encapsulated, vascularized, inferomedial, and extraconal mass in the left orbit. The mass displaces the eyeball and the inferior rectus muscle laterally and the medial rectus muscle cranially. (b) Axial T2 MRI scan shows the mass located in the anterior part of the left extraconal medial orbit.

Magnetic resonance imaging (MRI) showed an intraorbital, extraconal, oval, capsulated mass measuring 2.0 × 1.5 cm, located inferomedially in the anterior part of the left orbit. The medial rectus muscle appeared cranially displaced whereas the inferior rectus one was laterally displaced. After infusion of contrast medium the lesion showed a heterogeneous density reflecting hypervascularity (Figures 1(a) and 1(b)). The provisional radiologic diagnosis was benign tumour with high vascular component.

**Figure 2:**
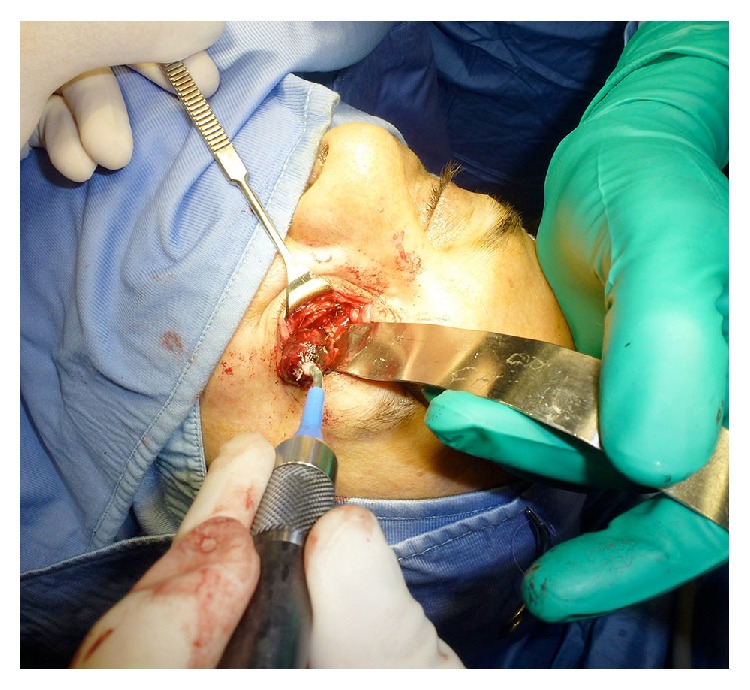
Intraoperative view: through transconjunctival approach and lateral canthotomy (swinging eyelid incision), we performed a subperiosteal orbital floor dissection. Then, the periorbita was incised inferiorly and medially. The well-encapsulated lesion was removed using a cryoprobe, pulling it laterally.

The patient underwent surgical operation at the Maxillofacial Surgery Unit of the University of Sassari for a benign left intraorbital mass of angiomatous nature. The tumour was completely removed in February 2012 through an inferior transconjunctival approach and a lateral canthotomy (Figure 2). The postoperative course was uneventful. Neither diplopia nor alterations of extrinsic ocular motility were observed. After 3 years of follow-up, the patient is free from disease.

**Figure 3:**
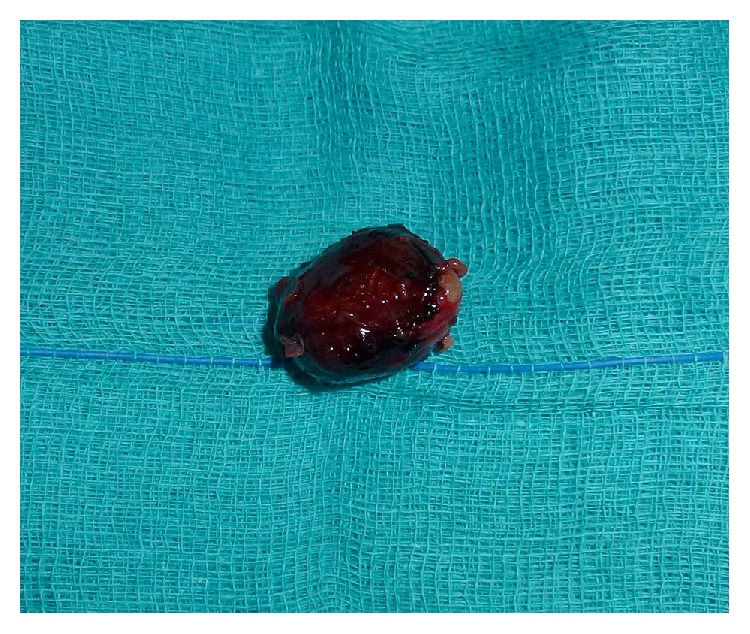
Macroscopy: the tumour mass appeared as a well-circumscribed nodule of 2.0 × 1.5 cm, brown in color.

At macroscopy the tumour appeared as a well-circumscribed nodule of 1.5 × 2.0 cm in diameter with a brown coloration (Figure 3).

**Figure 4:**
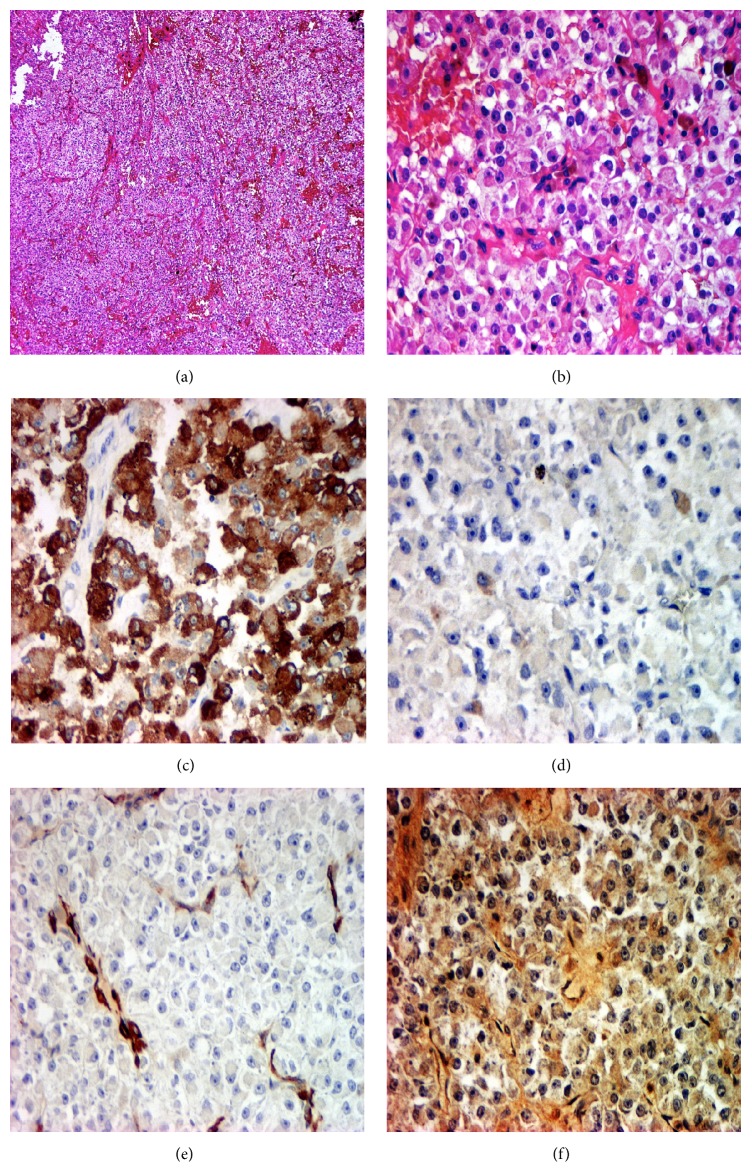
Histology: (a) trabecular architecture and nested growth pattern. HE, 40x. (b) Cell proliferation with an eosinophilic or clear granular cytoplasm; a prominent vasculature composed of delicate thin-walled capillaries was also appreciable. HE, 200x. (c) Immunohistochemical staining showing positivity for HMB45. 200x. (d) Immunohistochemical staining showed negativity for S100 protein. 200x. (e) Immunohistochemical staining for *α*-SMA showed slight staining in tumour cells. 200x. (f) Immunohistochemical staining showed focal positivity for Calponin. 200x.

Routinely processed paraffin-embedded tissue sections stained with haematoxylin and eosin (H&E) were evaluated. At histology the neoplasm showed a well-circumscribed, capsulated, expansive growth, formed by a cell proliferation arranged in vascular trabecular features or in solid cords. The cells were plump and large and showed an epithelioid phenotype with vesicular round nuclei, (small or large) nucleoli, and a low mitotic activity (2 mitoses in 50 HPF). The cells showed a clear or finely granular cytoplasm, sometimes containing a granular brown pigment. No necrosis was observed (Figures 4(a) and 4(b)).

The preliminary microscopic diagnosis was “angiomatous proliferation with epithelioid large cells resembling PEComa.” In order to better define the nature of the tumour, a panel of histochemical and immunohistochemical staining was performed, including Perls' Prussian blue and Schmörl techniques, CD 31, CD 34, Desmin, *α*-SMA, Vimentin, Cytokeratins AE1/AE3, CK8/18, Chromogranin-A (CgA), CD 10, HMB45, Melan A, and S100 protein.

The brown pigment was Perls' negative but Schmörl positive, so indicating melanin granules. At immunohistochemistry the cells were negative for all types of Cytokeratins, CD 31, and CD 34, while they were positive for HMB-45 (Figure 4(c)) but negative for Melan-A and S100 protein (Figure 4(d)). The cells were also negative for Vimentin, Desmin, and CgA. Slight positivity for *α*-SMA was observed (Figure 4(e)). The histological diagnosis was myomelanocytic tumour or melanocytoma belonging to the Perivascular Epithelioid Cell neoplasms (PEComas) group without true evidence of malignancy. Because of its infrequent location, the case was sent in consultation to the Verona Group [1] which confirmed the diagnosis. According to previous descriptions of immunohistochemical markers expression in PEComas arising at different sites [7], a second line of IHC was hence performed using antibodies against microphthalmia transcription factor (MiTF), transcription factor E3 (TFE3), and muscle marker Calponin. No immunostaining with MiTF and TFE3 antibodies was observed, whereas focal positivity for Calponin was appreciable (Figure 4(f)).

## 3. Discussion

In relation to histology and location of the tumour, different differential diagnostic hypotheses were considered: firstly a possible primary tumour as epithelioid haemangioma, paraganglioma, and epithelioid melanoma or secondly a metastasis from tumours with low malignant potential, such as renal cell carcinoma. All these possibilities have been reported in the literature [8–13].

The immunohistochemical procedures performed account for the strategy applied in order to clarify the diagnosis.

The negativity for CD 31 and CD 34 spoke against an angiomatous proliferation although these antibodies clearly stained a prominent vascular network of small vessels around the cells. Also Vimentin was negative, so excluding a mesenchymal derivation of the tumour. Furthermore, the absent expression of high and low molecular weight Cytokeratins did not support the hypothesis of a metastatic renal cell carcinoma and the CgA negativity was against the supposition of a paraganglioma. The positivity for HMB45 aroused the idea of melanoma, but the negativity for S100 protein and Melan A (Mart1) aborted this possibility too.

Therefore, considering morphology (large, plump cells with clear cytoplasm sometimes containing granular pigment Schmörl positive) and the immunohistochemical results (negativity for Cytokeratins, Vimentin, and *α*-SMA, and positivity only for HMB45) the diagnosis of Perivascular Epithelioid Cell tumour was considered the most plausible. Since the location of this tumour is unusual in the orbit, the case was sent for a second opinion to Bonetti and Zamboni group that confirmed the diagnosis.

In Table 1 we collected our case with those reported in the literature [5, 6] and we observed a quite complete histological similarity with only slight but not substantial responses to IHC, all consisting with the diagnosis of PEComa.

In our table we did not consider the two cases of ocular PEComas reported by Furusato et al. [14] since they were in extraorbital location, although these authors included in their review Iyengar et al.'s cases [5, 6]. However, the histological and immunohistological features were totally overlapping with the intraorbital PEComas.

Given the rarity of PEComas, clear criteria for malignancy have not been established so that the prognosis cannot be accurately determined. However, taking into account that none of the three cases of intraorbital PEComa had recurrences (up to 40 months of follow-up in our case), tumours with size below 5 cm and low mitotic rate without evidence of necrosis could be* bona fide* considered benign according to Folpe criteria [15].

## Figures and Tables

**Table 1:** Summary of main features of intraorbital PEComas.

Author	Sex	Age	Dimensions	Histology	IHC	Outcome
Iyengar et al. 2005 [5]	F	9	1.2 × 1.0 × 0.8 cm	Solid architecture with nests and trabeculae of epithelioid cells with abundant clear to eosinophilic cytoplasm. The uniform nuclei were oval to round with small nucleolus and dispersed chromatin. Within the lesion, a few cells had fine melanin pigment granules. There were infrequent mitoses. No evidence of necrosis	HMB45+ + + MART1− − − S100− − H&LMW CK− − EMA− − *α*-actin+ + − Desmin− − Calponin+ + VIM− CgA− −	No recurrence after 7 months of follow-up

Guthoff et al. 2008 [6]	M	54	1.5 × 1.0 × 1.0 cm	Solid trabecular tumour cells show intimate association with ramified vascular network. The tumour cells are large with a clear to eosinophilic granular cytoplasm and round to oval nuclei. Few cells contained melanin. Proliferation index (Ki 67) was very low (<1%)	HMB45+ + + MART1+ + + S100− − HMW CK− − *α*-actin− − Desmin− − VIM− CD 31− − CgA− − MiTF− −	No recurrence after 17 months of follow-up

Our case	M	47	2.0 × 1.5 cm	The neoplasm showed a well-circumscribed, capsulated, expansive growth, formed by a cell proliferation arranged in vascular trabecular features or in solid cords. The cells were plump and large and showed an epithelioid phenotype with vesicular round nuclei, small nucleoli, and a low mitotic activity (2 mitoses in 10 HPF). The cells showed a clear or finely granular cytoplasm, sometimes containing a granular brown melanin pigment. No necrosis was observed	HMB45+ + + MART1− − S100− − H&LMW CK− − CK8/18− − *α*-actin+ Desmin− − Calponin+ VIM− − CD 31− − CD 34− − CgA− − MiTF− − TFE3− −	No recurrence after 40 months of follow-up
